# A Descriptive Review on the Prevalence of Gastrointestinal Disturbances and Their Multiple Associations in Autism Spectrum Disorder

**DOI:** 10.3390/medicina56010011

**Published:** 2019-12-27

**Authors:** Radu Lefter, Alin Ciobica, Daniel Timofte, Carol Stanciu, Anca Trifan

**Affiliations:** 1 Center of Biomedical Research of the Romanian Academy, Iasi Branch, Romania, B dul Carol I, nr. 8, 700506 Iasi, Romania; radu_lefter@yahoo.com (R.L.); alin.ciobica@uaic.ro (A.C.); stanciucarol@yahoo.com (C.S.); 2 “Alexandru Ioan Cuza” University, Bd. Carol I, nr. 11, 700506 Iasi, Romania; 3 “Grigore T. Popa” University of Medicine and Pharmacy, 16 Universitatii Street, 700115 Iasi, Romania; ancatrifan@yahoo.com

**Keywords:** autism spectrum disorder, gastrointestinal disturbances, brain-gut axis, dysbiosis

## Abstract

*Background and Objectives:* Gastrointestinal disturbances have been frequently, but not unanimously, reported in autism spectrum disorder (ASD) individuals. Thus, digestive symptoms, such as constipation, diarrhea, abdominal bloating, and pain have been reported to correlate to the various maladaptive behaviors in ASD children, such as irritability, social withdrawal, stereotypy, hyperactivity, and even language regression. In this context, the present study provides an overview on the prevalence of the gastrointestinal (GI) disorders in ASD and the correlation between these and ASD symptoms and comorbidities and subsequently discusses the metabolic and microbiome factors underlying the effects of GI disorders in ASD. *Materials and Methods:* For our analysis of GI symptoms in children with ASD, we have searched peer-reviewed journals from 2005 to 2017 in PubMed databases that addressed the specificity of GI symptoms in ASD and included correlations of GI and ASD symptoms. The criteria for inclusion were clear quantitative mentioning of GI modifications, GI symptoms correlation with specific ASD symptoms or comorbidities, an appropriate methodology for defining ASD, and larger size samples. For this topic, only studies on human patients and original research were considered. A subsequent search in PubMed databases in journals from 2000 to 2017 we analyzed 13 articles on the mechanisms underlying the impact of GI dysfunctions in ASD, including gut microbial dysbiosis, immune reactivity, genetics, and altered neurotransmitters on the gut–brain axis. *Results:* In the 18 original research studies that we selected out of an initial 327 studies, despite the different methodology, a predominant 83% highlighted the increased prevalence of GI symptoms in ASD patients. Constipation was most frequently cited, appearing in 12 of the studies (80%), followed by diarrhea reports in eight studies (53%). The association between cognitive and behavioral deficits and GI disorders was suggested in certain groups of ASD individuals. *Conclusion:* The evidence presented so far by numerous studies seems to indicate that GI dysfunctions are of particular relevance in ASD, underlined by various abnormalities along the nervous connections between the central nervous system and the gut, such as impaired parasympathetic activity and increased endocrine stress response. Sufficiently large size samples and standardized methodology are required for future studies to clarify the complex interactions between GI disturbances and ASD symptoms.

## 1. Introduction

Autism spectrum disorder (ASD) is a complex disorder characterized by deficits in social communication and social interaction, restricted, repetitive patterns of behavior, interests, or activities, originating in the early developmental period, that are disabling for one’s life in terms of social, occupational, or other important areas of current functioning. Besides this specific triad of symptoms, a large number of children with autism exhibit various gastrointestinal (GI) symptoms, such as constipation, diarrhea, food allergies, malabsorption or maldigestion problems [1,2], more frequently than normal children. The prevalence of various GI disorders in children diagnosed with one of the autism spectrum disorders is reported to vary significantly from 9% to 91% [3]. A retrospective prevalence study on 14,000 autistic individuals under the age of 35, showed that bowel disorders held a significantly higher frequency than in the general population (11.7% vs. 4.5%), were one of the most frequent complications in the examined autistic population, besides schizophrenia or epilepsy, and were among the three co-morbidities, along with sleep disorders and epilepsy, that did not change significantly after the age of four [4]. In this context, the present paper aims to provide an overview on the prevalence of the GI disorders in ASD by reviewing previous studies and the correlation between these and ASD symptoms and comorbidities, and also to discuss the most important mechanisms facilitating the involvement of GI disorders in ASD, namely dysbacteriosis of the intestinal microbiota, inflammatory and immune reactions, hyperpermeability, and gut–brain connection. To conduct our analysis of GI symptoms among children with ASD, we searched PubMed databases in peer-reviewed journals for the period 2005–2017 for the main aim of obtaining evidence on the prevalence of GI in ASD children. Further, the specificity of GI symptoms in ASD and correlations of GI and ASD symptoms and other comorbidities were included. These data are transposed in Table 1. Secondly, we investigated gut microbiota dysbiosis and gut–brain axis involvement in ASD. Combinations of keywords were used: autism/autism spectrum disorder and gastrointestinal symptoms/disturbances and gut microbiota/microflora. The literature inquiry for our main topic yielded an initial 327 studies. Following the exclusion of incompatible titles and screening of abstracts, we selected 18 original research studies that, despite the broad range methodology to assess GI symptoms, were relevant for estimating prevalence of GI symptoms and correlations with ASD symptoms. The criteria for inclusion were clear quantitative mentioning of GI modifications, GI symptoms correlation with specific ASD symptoms or comorbidities, an appropriate methodology for defining ASD, and larger size samples. For this topic, only studies on human patients and original research were considered. Thirteen studies were referred to for the analysis of the mechanism underlying the impact of GI dysfunctions in ASD, gut microbial dysbiosis, immune reactivity, genetics, and altered neurotransmitters on the gut–brain axis.

## 2. Cognitive and Behavioral Deficits Related to Gastrointestinal Disturbances in ASD

The frequent GI symptoms in autistic children, such as abdominal pain, diarrhea, or constipation, seem to be related to maladaptative behavior. Thus, irritability, social withdrawal, stereotypy, hyperactivity, or inappropriate speech were found more often in autistic children with frequent GI symptoms than in children without frequent GI symptoms, during a large-scale CHARGE study conducted between 2003 and 2011 [5]. Similarly, a recent study by Ferguson et al. [22] in a sample of 340 children and adolescents with ASD found internalizing symptoms such as anxiety symptoms, withdrawn behavior, and depression to predict GI complaints in older children (age 6–18), and externalizing problem behavior, such as aggressive behavior associated with nausea in children aged 2–5.

Reviewing the data recorded in PubMed databases of the last 15 years regarding the correlations between the autistic cognitive deficiencies and GI abnormalities (Table 1), we have found some studies bringing relevant data in this matter, as presented in the table below.

In majority of the cited studies ASD subjects are diagnosed accordingly to valid criteria such as DSM (IV) and have clinical records. One study [11] does not have any direct ASD validation, but mentions recruitment was done on basis of a previous diagnostic done for a former study, whereas in Nikolov et al. study [8] a comprehensive assessment of clinical trials, medical history and screening questionnaire is carried out.

We were interested in studies with larger sample sizes of subjects diagnosed with ASD, i.e., over 100, still 27% of the total number of studies, had a medium size of 50 [5,6,11,19] only one study had a sample size of 15, but this was a metagenomic assay for bacterial gene sequences in ileo-cecal biopsies from ASD subjects [13].

More differences appear in the methods to diagnose GI symptomatology, and this was done by validated questionnaires that were administered to parents or caregivers, and only in a quarter of the studies [8,12,14,16,17] was a pediatric specialist also addressed. Interestingly, parental reports and gastroenterologist expertise were discordant in some instances [14] suggesting limited parental ability to discriminate variable GI symptoms. Out of the 18 studies analyzed, 15 (83%) reported increased prevalence of GI symptoms in the ASD patients, with constipation and diarrhea having the highest rates. Constipation/chronic constipation/functional constipation is most frequently cited, appearing in 12 of the studies reporting GI abnormalities (80%), followed by diarrhea reports in eight studies (53%). Other GI symptoms include abdominal pain (26%), altered stool patterns (20%), bloating or gas (20%), gastroesophageal reflux disease (13%), food selectivity and rejection (13%), and difficulty of swallowing (6%). Bresnahan et al., who found significantly three in four increased odds for constipation, and two in four for diarrhea in ASD children in a large longitudinal study over a 10-year period, also commented that these symptoms are more reliably recognized than other symptoms [18].

Further, 17% of the reviewed studies do not report any significant differences between ASD and children with typical development in terms of GI symptomatology [7,9,19].

The reason for the often-reported lack of significance of the GI symptoms in ASD may be the genetic and epigenetic heterogeneity of the autistic population that would predispose only certain groups to this type of disturbance, as well as the heterogenous methodology used by different studies. In this case, however, the three studies do not suffer any methodological divergence, nor do they lack sample size when compared to the other studies currently reviewed.

The cognitive deficits in autistic individuals have been clearly related to GI disorders in at least certain groups of autistic individuals.

As found in some studies, the odds of chronic constipation were found to be associated with both increased social impairment and a lack of expressive language or regression of language in children with ASD [5,14]. According to parental reports, children with language regression exhibited more frequently abnormal stool patterns and had an increased family history of celiac disease or inflammatory bowel disease [5].

Significant associations between functional constipation and rigid-compulsive and repetitive behavior, including tactile hypersensitivity/aversion were observed in a recent study with a sample size of 108 children with ASD [23]

In regard with the repetitive and ritualistic behavior, the reported significant incidence of constipation and food selectivity in autistic patients may be explained by the repetitive behavior and routines, endorsed by the notorious hyperreactivity to sensorial stimuli, that cause autistic children to choose and demand stereotyped diets that are usually low on fiber, fluids, and other food constituents [7]. These factors can lead to the avoidance of certain foods, textures, and tastes, such as low palatable vegetables in favor of the highly palatable, yet less nutritious snack foods [24]. A commonly reported GI disturbance in ASD is the gastroesophageal reflux disorder [16], which allows the insufficiently digested food, mixed with gastric juice, to return to the esophagus, causing heartburn, throat discomfort and regurgitation. One aspect concerning this manifestation regards the hand-mouthing repetitive behavior, that was determined to be a nonsocial behavior in ASD with the role of a negative reinforcement, especially when pain is present [25].

The negative correlation between anxiety, a common co-morbidity for autistic children and hypersensorial sensitivity, one of the core symptoms in ASD, was found to be significantly more prevalent in autistic children with chronic abdominal pain than in autistic children without abdominal issues [26]. Furthermore, diagnoses of anxiety disorder were identified as increasing the risk of GI dysfunction and impaired parasympathetic activity, and clusters of GI problems, anxiety, and autonomic dysfunction were suggested to characterize a subset of children with ASD [21].

Although not very numerous, there are several studies that suggest a factual correlation between the GI symptoms and severity of autistic signs, linked with abnormalities in the patterns of intestinal bacteria in children with ASD [11,13].

## 3. Gut Microbiota: Dysbiosis and Therapeutic Potential in ASD

The intestinal microbiota is composed by numerous bacterial species, that develop a mutualistic relation with the host and maintain a stable composition in normal conditions, forming certain human enterotypes, in which they are dominant [27]. Besides participating in the absorption of nutrients and immunity, these beneficial bacteria form a protective barrier, excluding potential pathogens. Alteration of environmental conditions leads to the development of pathogenic bacteria that disturb not only the digestive physiology, but also influence brain function and behavior.

Analysis of the bowel microbiota of ASD patients with GI disturbances, from stool samples or intestinal biopsy samples, have highlighted the occurrence of significantly higher numbers of the potential pathogens, such as Clostridia C. boltae, C. hystoliticum, C. perfringens [28,29,30], or Sutterella sp. [13], the latter normally present only in human faeces, as well as significant lower levels of Bifidobacterium [11].

Another aspect concerning the microbiota dysbiosis in ASD relates to the statistically significant negative correlation between the presence of autistic symptoms and bacterial richness. Significantly lower abundances of the genera Prevotella, Coprococcus and Veillonellacea, carbohydrate-degrading or fermenting bacteria, were observed by the analysis of bacterial rDNA gene sequences from stool samples of autistic children [31]. Tomova et al. [32] reported altered gut bacteria profiles in children with ASD as compared with neurotypical children, and also very strong association of increased amount of Desulfovibrio spp. with the severity of autistic restricted/repetitive behavior. A recent evaluation of gut microbiota profiles of ASD patients using DNA extraction and shotgun metagenomic analysis revealed alongside the decreased microbiota biodiversity and chronic state of peripheral low-grade inflammation coupled with increased levels of TNFα, TGFβ, and other immune factors, also decreased catabolism of 3,3 phenylpropionate speculated to ensue from the lower prevalence of *E. coli* [33]. Previous reports have shown that propionic acid may be associated with human neurodevelopmental disorders [34], and also the derivate 3-(3-hydroxyphenyl)-3-hydroxypropionic acid, usually related to Clostridium proliferation, has been found in higher concentrations in urinary samples of autistic children [35].

Administration of probiotic formulations, containing Lactobacillus and Bifidobacteria strains, well-known probiotic species, not only significantly reduced the digestive symptoms (flatulence, bloating) but also alleviated anxiety and depression in both human patients and in rats [36,37].

The manipulation of the gut microbiota, mainly by probiotic administration, has been proposed as a potential therapy for GI and behavioral symptoms in ASD, that could ameliorate communicative and stereotypic behaviors by an as yet unclear modulation along the gut–brain axis [38]. The beneficial effects of probiotics and probiotic formulations—most of them including the Lactobacillus and Bifidobacterium species [39]—have been demonstrated in recent studies, both in animal models [36,38] and in autistic subjects [5,11], in relieving the GI disturbances, restoring the gut barrier, modulating the gut microbiota, and stimulating the immune system [40,41].

Although this novel therapeutic approach has the appeal of a providential/promising cure, the complex dynamics of the gut–brain axis are not fully understood and the conclusions drawn are often controversial. In this regard, as Li and Zhou observed in a recent review [40], the gluten-free or casein-free diets which were reported in some studies to improve the behavioral symptoms of ASD children [42,43], were found ineffective in others [44,45].

Also promising for rebalancing gut microbiota and alleviating GI (and ASD) symptoms is the microbiota transfer therapy (MTT), a modified protocol of fecal microbiota transplant based on the chronic administration of doses of standardized human gut microbiota prepared using stool from healthy individuals as starting material. MTT has been pioneered by Kang et al. in a recent open-label clinical trial involving 18 children with ASD leading to increases in bacterial diversity and 80% reduction of GI symptoms at the end of the 10 weeks treatment including the symptoms of constipation, diarrhea, indigestion, and abdominal pain [46]. Moreover, clinical assessments showed a slow but steady improvement in core ASD symptoms, and the improvements were sustained after an eight-week follow-up observation period [46]. Remarkably, in a recent follow-up with the same 18 participants, two years after treatment, GI benefits were mostly maintained and autism-related symptoms improved even more [47].

Despite these promising results, the probiotics or fecal microbiota transplants are in an incipient stage and their significance in the treatment of children with ASD must be further assessed following a robust uniform methodology and on large group samples.

Several recent studies [48] show that microbiome of normal and ASD subjects differ in an intriguing way from country to country. Thus, a series of studies on the faecal microbiota sampled from traditional foraging communities in Tanzania [49] and rural agrarian communities in Malawi [50], Burkina Faso, and Amerindians from Venezuela [51] showed, as compared to the samples of European or USA urban controls, significantly higher levels of microbial abundance and biodiversity. The subjects were in early infancy as well as adulthood. These data indicate that, on one hand, the gut microbiota composition is highly dependent on the geographic and dietary factors, and, on the other hand, the specific Western diet, low in fiber and high in simple carbohydrates, favors a typical “hyper-Westernized” reduced gut microbiota in numbers and diversity—such as the decreased abundance of the bacterial genera Prevotella [52]. If not all, then at least certain subtypes of the ASD population, is particularly vulnerable to such environmental-dietary influence due to the innate predisposing genetic abnormalities.

## 4. Intestinal Hyperpermeability and Immune Reactivity

Increased intestinal permeability and malabsorption have been reported as GI issues in ASD by several studies, with a 10-fold increased prevalence as compared to healthy individuals [53]. There are mixed results on the significance of dietary factors, including gluten and casein relative to the manifestation of GI symptoms and in particularly to stress-responsive cytokines and immune markers. Lau et al. [54] reported that children with ASD and GI adverse manifestations displayed an increased immune reactivity to gluten, even when compared to autistic patients without GI symptoms, thus linking the response of anti-gliadin antibodies and GI symptoms. Gluten-casein-free diet were signaled to significantly lower the abnormal high intestinal permeability values in subgroups of ASD patients [55] and even resolve the GI symptoms and progressively ameliorate ASD symptoms in a clinical case diagnosed with severe autism [56].

However, in a very recent study, Ferguson et al. [57] reports no significant correlations between dietary factors including consumption of gluten or casein, and GI symptoms, reporting instead a strong relationship between stress reactivity and GI problems in a sample of 120 ASD individuals [21,57]. Also, there are other recent studies that did not find an association between dietary gluten/milk, intestinal permeability, and behavioral changes in subjects with ASD [19,58], which may be indicative for the variability of the autistic phenotype.

Entering of the insufficient digested alimentary particles or certain toxins caused by an increased intestinal permeability, activates the release of antibodies, triggering inflammatory reactions and a subsequent decrease in the levels of immunoglobulins, causing dysbiosis, the proliferation along the intestinal tract of microbial pathogen species, such as clostridia, or fungi.

Given the fact that the prevalence of ASD has continuously and steeply increased, especially in the last decade in Westernized countries [59], a question arises whether this can be caused by an increase of the incidence of etiologic factors related to the increasing modernization and implicit socio-economic factors, or rather by the more accurate diagnosis methods, or public awareness [60]. Intestinal hyperpermeability and immune responses can also be related to the low abundance of the beneficial bacteria and the increased prevalence of potentially pathogenic microorganisms.

## 5. Genetic Abnormalities Favoring GI Comorbidities in ASD

The genetic predisposition in the autistic etiology is undeniable, and proven by the estimated heritability of ASD symptoms, more than 90% for monozygotic twins and only 53% for dizygotic twins [61,62]. However, the multiple subtypes within the larger frame of ASD are not likely to have one single common genetic cause. It is rather epigenetics, as the result of the interaction between the genes and environmental factors, to provide the most sensible explanation for the diversity of phenotypes.

Several connections have been found between genetic abnormalities and the GI comorbidities accounted in autistic cases that may explain the significance of these clinical features for the disorder. Thus, a single nucleotide polymorphism of the c-Met proto-oncogene, that encodes MET receptor tyrosine kinase, which causes MET hypofunction, was consistently found in a subset of ASD individuals with comorbid GI diseases [38]. The MET receptor mediates the effects of the hepatocyte growth factor in neuronal outgrowth and dendritic morphology, but also in immune function and intestinal epithelial development [38,63,64,65]. Another susceptibility gene for ASD that may be associated to GI dysfunctions is the *CHD8*, which is involved in chromatin remodeling and histone deacetylation modulation [66]. Li and Zhou mention in a recent review a higher frequency of constipation in children with *CHD8* mutations than in children without the mutation [40], while in zebrafish the mutations of the *CHD8* gene induce features of the human phenotype such as macrocephaly (often reported in ASD) and also impairs GI motility affecting the neuronal colonization of the GI tract [67]. The 5-HT transporter (SERT) gene (*SLC6A4*) coding variants, which cause the hyperserotonemia phenotype subtype and represent one of the most robust biomarkers for ASD [68] may also play a role in the GI disturbances since endocrine cells of the GI tract produce most of the body’s serotonin and intestinal enterocytes are known to express SERT [38,69]. In regard to this hypothesis, Marler et al. [70] explored the relationship between GI symptoms and 5-HT in a group of 82 children and adolescents with ASD and confirmed the frequency of hyperserotonemia and constipation in ASD. Interestingly, serotonin levels were correlated with some lower tract GI symptoms, including pain, stool retention, and large bowel movements but not with functional constipation [70], which leaves unclear whether these associations are specific only to ASD.

## 6. Neurotransmitters Abnormalities along the Brain–Gut Axis

This intrinsic circuit that reciprocally connects (via the vagus nerve and the enteric nervous network) the GI tract and the central nervous system is involved in the regulation of digestive processes (including appetite and food intake) [71], but also in modulating a number of cerebral complex behaviors, including emotional and cognitive behavior, anxiety and stress [72]. Interestingly, GI symptoms (functional constipation) as well as lower intelligence index and inappropriate speech were significantly associated with increased endocrine stress response in ASD children [73]. In this context, the enteric microbiota, as an essential component within the gut environment, clearly emerges as a principal factor in influencing brain functioning and behavior [72]. Neurotransmitter levels, such as those of serotonin (5-HT), glutamate, or γ-aminobutyric acid (GABA), which are altered in ASD, may fluctuate as a result of gut microflora modifications. Altered gut serotonin signaling is suggested as a mechanism in the GI affections observed in ASD, such as the inflammatory bowel disease or the irritable bowel syndrome, as increased 5-HT is a very strong proinflammatory and immunomodulatory agent for the intestinal mucosa [3]. Serotonin, which is present in the gut over 90% of its total quantity, not only regulates mood, appetite, or sleep, but is involved in cerebral cognitive functions, such as memory and learning [38]. Also, it is well known that abnormal serotonin system functioning is involved in neurodevelopment processes and is a major factor in the pathophysiology of ASD, as it influences neurogenesis and/or neuronal removal, neuronal differentiation, and synaptogenesis [74]. In the gut, serotonin is involved in the digestive processes and its reuptake is carried out mainly by the serotonin re-uptake transporter (SERT). American researchers at Vanderbilt University identified that the most common genetic SERT mutation (*SERT Ala56*) appears in the genes of hyperserotonemic autistic children. The manipulation of this gene in mice led to autistic models of mice that exhibited communication delays and repetitive behaviors similar to those observed in children with autistic spectrum disorders [68] and to significant abnormal regulation of GI motility and perturbed enteric neuronal development and mucosal growth [75]. Further investigations showed that *SERT Ala56* associated deficits were prevented when animals were treated during embrionary development with a 5-HT4 agonist, which may indicate that a defective 5-HT pathway caused by early life insults underlie behavioral and GI symptoms in ASD [75].

With respect to γ-aminobutyric acid (GABA), the main inhibitory neurotransmitter, essential in maintaining the balance excitation-relaxation, which is deficient in ASD, it was suggested that human intestinally derived strains of the beneficial Lactobacillus sp. and Bifidobacterium sp. are very efficient GABA producers [76]. In genetically manipulated mice, with altered GABA receptor expression, the chronic dietary ingestion of Lactobacillus rhamnosus mediated the expression of GABA in the prefrontal cortex, amygdala, and locus coeruleus, and reduced stress-induced corticosterone, alleviating anxiety or depressive behavior [77]. Another mechanism that microorganisms may deploy within the gut-brain axis could be axon injury or stimulation (neuroinflammation) by bacterial cytotoxins and cytokines, which may result in the disruption of the blood-brain barrier, changes in neurotransmitters secretion or of the stomach and intestinal microbiota [78]. Changes in the GI microenvironment may influence the activation of the vagal afferent pathways that link the mucosa of the intestinal epithelium and the brainstem, via the nucleus solitarius path. Goehler et al. suggested that among the multiple mechanisms that ensure the communication from the immune system to the brain, the peripheral sensory neurons (mainly the vagal sensory neurons) are the first to activate early on in an infection [79]. By inducing local infection in the gut of mice with Campylobacter jejuni, a significant increase of the neuronal marker c-Fos expression in the neurons of the vagal sensory ganglia and the nucleus of the solitary tract, the primary sensory relay nucleus for the vagus, was observed prior to any immunosensory signaling such as elevated levels of circulating pro-inflammatory cytokines [80]. The altered microbiota can exert a direct metabolic action against the neural processes through metabolites synthesized in dysbiotic conditions. Thus, the previously mentioned HPHPA which depletes brain catecholamines and causes symptoms of ASD, such as stereotypical behavior, hyperactivity, and hyper-reactivity in experimental animals, appears in higher concentrations in the urine of children with ASD and is produced by Clostridium species [81]. The bacterially mediated p-cresol found in urine samples of autistic patients indicates a reduced capacity to sulfonate certain phenolic amines, toxic for the CNS, that are potentially exacerbating the autistic behavior [82]. The 4-ethylphenylsulfate, which is produced by several gut bacteria, induces autistic features and anxiety-like behavior when injected in mice, and, furthermore, was found to be 46-fold increased in autistic maternal immune activated mice [52,83]. Treatment with the commensal Bacteroides fragilis ameliorated autistic-like behavior in maternal immune activated mice models, supposedly by decreasing of indolepyruvate which is produced by gut microbes in dysbiotic conditions [83]. Interestingly, this treatment ameliorated the communicative behavior and stereotypical manifestations, but not the sociability deficits, suggesting that different behavioral traits in ASD are sustained by different systemic circuits and may have a relative causal autonomy [83,84].

## 7. Limitations of the Studies to Date and Suggestions for Future Research

Currently, the studies on the role of the GI disturbances in the etiology and development of ASD suffer from the lack of an established methodology, which leads to numerous differences and inconsistencies. Firstly, the group samples of children with ASD should be sufficiently large to counter the heterology of the disorder, as it is possible that the abnormal GI microbiome only occurs in certain subgroups of ASD children or at certain phases in the course of their condition [29]. Heterogeneity inside the groups, both the control group and the ASD group, need to be reduced as possible in terms of age, gender, diet, oral antibiotic use, prebiotics, probiotics, and other medical conditions [52].

Secondly, a major issue concerns the different assessment methods in evaluating GI symptoms in children with ASD, as clinical reports and questionnaires need to rely on parental observations and interpretations, a source of heterogeneity, as Hollingue et al. [85] observed, given the variability of parental perceptions, which may explain the large variability in the prevalence of GI problems in ASD (9–91%) [3]. Future studies must aim to consistently use a standardized method of assessing GI symptoms with clearly defined criteria, such as the recent 2016 Rome IV criteria for functional GI disorders. Furthermore, reliable questionnaires should be developed to efficiently assess GI symptoms in the ASD frame, such as, for example, a two stage ATN derived questionnaire, separately completed by caretakers and pediatric gastroenterologists, with a higher predictive value [86].

Thirdly, another critical limitation for the current research, as McDonald highlighted in a recent review, resides in the lack of an encompassing reference data set of the digestive microbiota samples [87]. Such a data set would help in standardizing the methodological approaches and answer to the technical limitations and constraints currently met by the researchers, concerning the type and size of samples to be analyzed, the different methods of characterizing the microbiota, and the different analysis and statistics methods. Among the best known databases are the Human Microbiome Project, which aims to characterize microbial communities found at multiple human body sites and identify correlations between changes in the microbiome and human health [88], or the Earth Microbiome Project [89], intended to map the uncultured microbial diversity across 200,000 environmental samples. These are dynamical projects, which can be continuously improved by ongoing researches, an advantage that could turn into a disadvantage in some cases when combining dissimilar datasets [87]. Developing such databases will permit using the data on gut microbiota and brain–gastrointestinal interactions in the larger context of the host genetics and epigenetics variability, and may offer a new view on the etiology of ASD [90].

## 8. Conclusions

GI abnormalities such as abdominal pain, diarrhea, constipation, gastroesophageal reflux, and food selectivity have been described in autistics, but a clear and convincing link of these symptoms to ASD has not yet been found. However, the evidence presented so far by numerous studies seems to indicate that GI dysfunctions are of particular relevance, underlined by various abnormalities along the nervous connections between the central nervous system and the gut, such as impaired parasympathetic activity and increased endocrine stress response. In this way, the most encountered GI abnormalities, such as constipation, abdominal pain, diarrhea, gastroesophageal reflux, and food selectivity, were found in correlation with typical manifestations, such as social withdrawal, stereotypy or repetitive and ritualistic behavior. Further, clusters of GI problems, anxiety, and autonomic dysfunction have been suggested to characterize a subset of ASD population.

Also, the microbiota seems to have a very large role on determining the brain’s fate during the first period of life, as the disruption of the normal benefic bacteria, due to various environmental insults, among which the genetic and immunologic are prevalent, may lead to dysbiosis in favor of more resistant pathogenic neurotoxin-producing bacteria. These disturb not only the GI function, but also influence brain function and behavior, and altered gut bacteria profiles in children with ASD have been found together with increased severity of autistic restricted/repetitive behavior.

Moreover, the immune inflammatory reactions reported by some studies to occur more frequently in ASD as a result of incomplete digested alimentary particles, may be potentially leading to nutritive deficiencies and specific allergic sensitivities, such as gluten or casein sensitivity. At the same time, the disruption of the immune system facilitates the development of pathogenic bacterial strains and dysbiosis, causing further GI complications and, through their metabolic products, the alteration of various neurologic processes. Although specific diets were shown to alleviate the behavioral deficits in some autistic cases, the specific ways of action and impact of these gut microbiota manipulating interventions on the ASD symptoms are not yet clearly delineated nor universally accepted. However, dysbiosis has been confirmed by many studies to alter the CNS processes through bacterial metabolites such as hydroxypropionic acid or p-cresol and exacerbating the autistic behavior, hence the promising results of probiotics and faecal microbiota transfer therapy in improving GI and ASD symptoms. Mechanistically, GI symptoms in ASD should be considered linked to the multiple paths of the gut–brain axis, and besides the microbial and immune component, the various GI manifestations are related to the autonomous nervous system affecting the parasympathetic tone and stress responsiveness as well as to abnormal dynamics of neurohormones, such as serotonin or GABA. The complexity of these interactions and also the numerous controversies to date call for future extensive research to be performed on sufficiently large sized groups after a standardized method of assessing GI symptoms within the ASD frame.

## Figures and Tables

**Table 1:** Prevalence of gastrointestinal (GI) symptoms and correlations with autism spectrum disorder (ASD) symptoms.

Author	Study Type	Sample Size (ASD/Control)	Overall Prevalence of GI Symptoms	Specificity of GI Symptoms in ASD Cases (vs. Control)	Correlations between GI Symptoms and Autistic-Like Symptoms	Other Observations
Parracho et al., 2005 [5]	Faecal bacterial populations assessment by Fluorescence in Situ Hybridization analysis	58 children with ASD/a non-ASD sibling group (N = 12)/an unrelated healthy group (N = 10). Age between 3–16 years	ASD group: 91%. the sibling group: 25% the unrelated group: no acknowledged gut problems. the ASD patients were specifically chosen due to known GI issues	diarrhoea 75%, excess wind 55%, abdominal pain 46.6%, constipation 44%, abnormal faeces 43.0%.	Significantly more frequent GI problems in ASD group vs. controls (*p* = 0.005) may indicate an association between GI symptoms and ASD.	Significantly higher levels of clostridia (*p* = 0.001) in ASD group vs. unrelated healthy group
Valicenti-McDermott et al., 2008 [6]	Cross-sectional study comparing the lifetime prevalence of GI symptoms	50 children with ASD/50 with other development disorders (DD)/50 with typical development (TD) Mean age: 7.6 years	Lifetime GI symptoms in 70% ASD group 28% TD group (*p* < 0.001) 42% DD group (*p* = 0.03).	Chronic constipation 44% (vs. 16% TD) *p* = 0.23) abnormal stool pattern 18% (vs. 4% TD, *p* = 0.039) food selectivity 60% (vs. 22% TD, *p* = 0.001)	In the multivariate analysis, ASD (adjusted odds ratio (OR), 3.8; 95% confidence interval (CI), 1.7–11.2) and food selectivity (adjusted OR, 4.1; 95% CI, 1.8–9.1) were associated with GI symptoms.	Children with ASD have a higher rate of GI symptoms than children with either typical development or other DDs.
Ibrahim et al., 2009 [7]	Long term population-based study of the incidence of GI symptoms in children with ASD and age- and gender-matched controls.	121/242 Mean age: 18 years	No significant association of ASD cases status and overall incidence of GI symptoms	Significant differences in the cumulative incidence by age 20 for: constipation 33.9% (vs. 17.6%, *p* = 0.003) feeding issues and food selectivity 24.5% (vs. 16%; *p* = 0.009).		
Nikolov et al., 2009 [8]	Clinical trials; assessment of GI disorders by medical history and screening questionnaire	172 children with ASD, part (88%) of a well-characterized sample of children with PDDs. Mean age: 8.3 years.	39 (22.7%) with moderate/severe GI symptoms	Primarily constipation (N = 14) and diarrhoea (N = 7)	Significantly higher scores for irritability, anxiety, and social withdrawal (*p* = 0.001) in the ASD-GI group, compared with ASD-no GI problems group.	
Sandhu et al., 2009 [9]	ALSPAC cohort (12,984 children) study; periodic questionnaires on ASD children’s stool patterns and gut symptoms	78 ASD group/12 906 the remaining children in the cohort	No major differences between the ASD and control group during the first 3.5 years of life (stool pattern, diarrhea, constipation, bloody stools or abdominal pain)	Slight increase in stool frequency at 30 and 42 months for the ASD group 57.6% (N = 38) vs. 44.0% (N = 4396) *p* = 0.039		
Krigsmann et al., 2010 [10]	Chart review, diagnostic subsequent ileocolonoscopy in children with ASD and ileocolonic disease.	143 children with ASD/developmental disorder patients, with chronic GI symptoms		Diarrhea 78%, abdominal pain 59%, constipation 36%.	Significant association between ileo and/or colonic inflammation or lymphonodular hyperplasia (LNH) and onset of the developmental disorder	Ileal and/or colonic LNH present in 73.2% of the sample group
Adams et al., 2011 [11]	Bacterial and yeast identification from stool samples of children with ASD and GI reported problems	58 ASD/39 healthy typical children. The ASD group was divided in 2 subgroups, with high and low GI problems	Significantly greater GI symptoms in ASD group, as the control group was specifically chosen with no GI problems.		Very strong correlation between GI and autistic symptoms: as evidenced by autism severity test scores between the ASD-high GI and ASD-low GI groups (+103% difference in speech/language/communication, and +53% in sociability test) *p <* 0.001	Significant lower levels of *Bifidobacterium* (−45%, *p* = 0.002), slightly lower levels of *Enterococcus* (−16%, *p* = 0.05) in ASD group compared with control
Wang et al., 2011 [12]	Large registry-based study in-home structured, retrospective medical history interviews	589 subjects with idiopathic, familial ASD/163 unaffected sibling controls	In ASD group: N = 249 (42%) in control group: N = 20 (12%) (*p* < 0.001).	Most common Gl problems in the ASD group: constipation N = 116; (20%) and chronic diarrhoea N = 111 (19%).	Increased ASD symptom severity was associated with higher odds of GI problems	
Williams et al., 2011 [13]	Carbohydrate digestion genes expression assays and analysis of bacterial 16S rRNA gene sequences from ileo-cecal biopsies	15 children with ASD and GI diseases/7 controls with GI diseases	Prevalence of specific GI symptoms was similar in ASD-GI and Control-GI groups	Diarrhoea 80% (vs. 71%) changes in stool frequency 87%; (vs. 71%) more frequent bloating in ASD-GI: 60% (vs. 29%)	87% of AUT-GI subjects had behavioral regression; 73% language loss.	Significantly higher levels of Clostridiales for the ASD-GI subgroup for which the GI symptoms occurred before or at the same time as the onset of ASD
Gorrindo et al., 2012 [14]	Parental interviews and pediatric gastroenterological evaluation of children with ASD and GI disorders (GID) between 2009 and 2011	121 children, in three groups: ASD-GI (n = 40); ASD-only (n = 45); GID-only (n = 36) Mean age: 10.8–12.4 years.	parental report and clinical diagnosis of any GID were highly concordant (92.1%).	Functional constipation was the most common in the ASD-GI group (85.0%).	Strong association between functional constipation and increased social impairment (*p* = 0.02), and lack of expressive language (*p* = 0.002) in the ASD-GI group	Presence of GID in ASD-GI group was not associated with distinct dietary habits or medication status
Mannion and Leader, 2013 [15]	Self-constructed demographic questionnaire to assess sleep problems and GI symptoms in ASD patients	89 children and adolescents with ASD/no control Mean age: 9 years	79.3%—at least one GI symptom within the last three months.	Most common GI symptoms were: abdominal pain in 51.7%, constipation in 49.4% of participants.	GI symptoms were found to be significant predictors for the persistent sleep problems (encountered in 80.9% ASDs children)	Increased prevalence of a comorbid disorder from 46% to 78% if intellectual disability is present
Chaidez et al., 2014 [16]	Data analysis, based on parent reports, from a CHARGE Study population-based sample nearly 1000 children 2003–2011	499 children with ASD/324 with typical development (TD)/137 with developmental delay (DD) Age between: 24 and 60 months	ASD and DD groups were three time more likely to present at least one frequent GI symptom as compared to TD children	In both ASD or DD: frequent constipation 15.5%, 15.8% (vs. 3.5% in TD) diarrhoea (13%, 6.1% vs. 1.6%, difficulty swallowing (4.2%, 4.6%, vs. 0.3%) food allergies, restrictions, and food dislikes were highest in children with ASD	In the ASD group, irritability, social withdrawal, stereotypy and hyperactivity were significantly higher in children with frequent occurrences of abdominal pain, gaseousness, diarrhoea and constipation as compared to the ASD children with no frequent GI.	
Kang et al., 2014 [17]	Clinical study of the GI dysfunctions in a cohort of children with ASD; endoscopic and colonoscopic evaluation	164 children with ASD	49%—one or more chronic GI complaints	Diarrhoea 22%; constipation 26%; bloating and/or gaseousness 13%; vomiting or gastroesophageal reflux problems 10%	Significant correlation of GI dysfunctions with sleep disorders and food intolerance, but not with irritability or aggressiveness	Inflammation of the gut was in 6 of the 12 subjects who underwent endoscopic and colonoscopic evaluations
Bresnahan et al., 2015 [18]	Large prospective cohort study during a 10-year period; maternal reports age 18- and 36-month questionnaires.	195 children with ASD/4636 children with developmental delay (DD)/4095 control group	Significantly higher prevalence of GI symptoms in the ASD group in either the 6–18 months or the 18–36 months old age period	Significantly increased odds of constipation (*p* < 0.001) and food allergy/intolerance, diarrhoea (*p* < 0.001), constipation (*p* < 0.01) compared with children with TD.		
Pusponegoro et al., 2015 [19]	Observational statistical cross-sectional study	48 children with severe ASD/111 with mild ASD/66 control	No significant differences between groups for the GI symptoms (about 20% in all groups)		The results suggest that maladaptive behavior in children with ASD is not associated with impaired intestinal permeability	significant enterocyte damage in the severe ASD group as compared to the other groups
Fulceri et al., 2016 [20]	A case–control study based on parental report investigating the behavioral problems and GI symptoms in 230 preschoolers	115 ASD with (ASD/GI+) or without GI (ASD/GI-) symptoms, age-matched control group of 115 peers with (TD/GI+) or without (TD/GI−) GI symptoms	Significant higher GI symptoms prevalence in the ASD group (37.4%) versus control (14.8%)	Most frequent GI symptoms present both in ASD and in TD groups, but more severe in ASD patients: constipation 15% (vs. 3.5%), *p* = 0.003 food refusal 27% (vs. 10.4%) and abdominal pain 14% (vs. 3.5%).	Significantly increased autistic behavior in the ASD/GI+ group vs. ASD/GI−: anxiety, somatic complaints, externalizing problems. No significant behavioral differences between the TD/GI+ and TD/GI−di	GI symptoms should be accurately assessed in ASD children with anxiety and/or externalizing behavioral problems.
Ferguson et al., 2016 [21]	Questionnaire based study and exploratory analyses of stress response	120 ASD diagnosed individuals with or without GI symptoms Age between 6 and 18 years	Higher prevalence of lower GI tract symptoms in contrast to reduced upper GI tract problems	Significantly increased functional constipation (42.5%) and lower abdominal pain associated with irritable bowel symptoms (9.2%)	Significant association between constipation vs. impaired parasympathetic functioning and constipation vs. regression/loss of skills and anxiety	anxiety disorders in ASD may increase risk of GI symptoms by enhanced stress response
Ferguson et al., 2016 [22]	Retrospective analysis based on parent and self-reports and statistical analysis of multiple variables	340 children and adolescents with ASD parsed into two age groups younger (ages 2–5) and older (ages 6–18)	High prevalence of GI symptoms	Constipation (65%), stomach aches or stomach pain (47.9%), nausea (23.2%), diarrhoea (29.7%)	Association between internalizing symptoms (anxiety and GI symptoms in older group and externalizing behavior and GI symptoms in the younger group	GI disorders and behavioral responses have different relationships at different ages in ASD
